# Root Extract of a Micropropagated *Prunus africana* Medicinal Plant Induced Apoptosis in Human Prostate Cancer Cells (PC-3) via Caspase-3 Activation

**DOI:** 10.1155/2022/8232851

**Published:** 2022-01-25

**Authors:** Richard Komakech, Nam-Hui Yim, Ki-Shuk Shim, Haiyoung Jung, Jae-Eun Byun, Jun Lee, Denis Okello, Motlalepula Gilbert Matsabisa, Joseph O. Erhabor, Omolola Oyenihi, Francis Omujal, Moses Agwaya, Yong-goo Kim, Jeong Hwan Park, Youngmin Kang

**Affiliations:** ^1^Herbal Medicine Resources Research Center, Korea Institute of Oriental Medicine (KIOM), 111 Geonjae-ro, Naju-si, Jeollanam-do 58245, Republic of Korea; ^2^University of Science & Technology (UST), Korean Convergence Medicine Major KIOM, 1672 Yuseongdae-ro, Yuseong-gu, Daejeon 34054, Republic of Korea; ^3^Natural Chemotherapeutics Research Institute (NCRI), Ministry of Health, P.O. Box 4864, Kampala, Uganda; ^4^Korean Medicine Application Center, Korea Institute of Oriental Medicine, 70 Cheomdan-ro, Dong-gu, Daegu 41062, Republic of Korea; ^5^Korea Institute of Oriental Medicine (KIOM), 1672 Yuseongdae-ro, Yuseong-gu, Daejeon 34054, Republic of Korea; ^6^Immunotherapy Research Center, Korea Research Institute of Bioscience and Biotechnology (KRIBB), 125 Gwahak-ro, Yuseong-gu, Daejeon 34141, Republic of Korea; ^7^Department of Functional Genomics, Korea University of Science and Technology (UST), 113 Gwahak-ro, Yuseong-gu, Daejeon 34113, Republic of Korea; ^8^Department of Biochemistry, School of Life Sciences, Chungbuk National University, Cheongju 28644, Republic of Korea; ^9^IKS Research Group, Department of Pharmacology, Faculty of Health Sciences, University of the Free State, Bloemfontein, 9301, Free State, South Africa; ^10^Phytomedicine Unit, Department of Plant Biology and Biotechnology, University of Benin, PMB 1154, Benin City, Nigeria; ^11^KM Data Division, Korea Institute of Oriental Medicine (KIOM), 1672 Yuseongdae-ro, Yuseong-gu, Daejeon 34054, Republic of Korea

## Abstract

Prostate cancer is one of the major causes of cancer-related deaths among men globally. Medicinal plants have been explored as alternative treatment options. Herein, we assessed the *in vitro* cytotoxic effects of 70% ethanolic root extracts of six-month-old micropropagated *Prunus africana* (PIR) on PC-3 prostate cancer cells as an alternative to the traditionally used *P. africana* stem-bark extract (PWS) treatment. *In vitro* assays on PC-3 cells included annexin-V and propidium iodide staining, DAPI staining, and caspase-3 activity analysis through western blotting. PC-3 cells were exposed to PWS and PIR at different concentrations, and dose-dependent antiprostate cancer effects were observed. PC-3 cell viability was determined using CCK-8 assay, which yielded IC_50_ values of 52.30 and 82.40 *μ*g/mL for PWS and PIR, respectively. Annexin-V and PI staining showed dose-dependent apoptosis of PC-3 cells. Significant (*p* < 0.001) percent of DAPI-stained apoptotic PC-3 cells were observed in PWS, PIR, and doxorubicin treatment compared with the negative control. PWS treatment substantially elevated cleaved caspase-3 levels in PC-3 cells compared with the PIR treatment. These results provide evidence for the antiprostate cancer potential of PIR and sets a basis for further research to enhance future utilization of roots of young micropropagated *P. africana* for prostate cancer treatment as an alternative to stem bark. Moreover, micropropagation approach may help provide the required raw materials and hence reduce the demand for *P. africana* from endangered wild population.

## 1. Introduction

Prostate cancer is one of the most common nonskin cancers, affecting 9%–11% of men worldwide [1, 2], and one of the leading causes of cancer deaths in men [3–6]. Prostate cancer develops due to uncontrolled prostate cell division, which in turn causes abnormal cell growth and spreads to other parts of the body [1]. During the initial stages, prostate cancer is androgen-dependent, and trans-activation of target genes occurs due to androgens binding to the androgen receptor [7, 8]. Under normal conditions and in the case of cancer androgens and androgen receptor-mediated signaling play pivotal roles in prostate functioning and development [9]. Therefore, downregulation of androgens through antiandrogenic agents is vital in prostate cancer treatment [6, 7, 10]. Typically, this cancer is treated using numerous conventional methods, including radiation therapy, surgery, hormone therapy, and cryosurgery [11]; however, these methods are frequently associated with various adverse side effects [12]. Thus, other avenues with fewer adverse effects are being continuously tested for effective treatment of prostate cancer. One such approach is the use of medicinal plants [13–15].

Many medicinal plants such as African cherry (*Prunus africana* (Hook f.) Kalkman; Rosaceae family) exert strong antiprostate cancer effects [9]; further, in previous ethnomedicinal studies, *P. africana* bark decoction was used to treat cancers, including prostate cancer [1, 16]. Many scientific studies have confirmed the significant antiprostate cancer effects of *P. africana* stem bark, whereby its use as an antiprostate cancer agent has in fact already been patented [17]. In an *in vivo* study, transgenic adenocarcinoma of the mouse prostate (TRAMP) mice fed on *P. africana* (synonym *Pygeum africanum*) showed a significant reduction (*p*=0.034) in prostate cancer incidence compared with casein-fed mice [18]. Similarly, in an *in vitro* study, the bark extract of *P. africana* was observed to induce about 50% growth inhibition of human prostate cancer (PC-3) and also induced significant apoptosis in the PC-3 cell line [18]. PC-3 is one of the main cell lines in *in vitro* studies on human prostate cancer [19].

The anticancer activity of the stem bark of *P. africana* has been attributed to numerous novel bioactive compounds, including *β*-sitosterol, ferulic acid, *β*-sitosterol-3-O-glucoside, lauric acid, oleanolic acid, ursolic acid, atraric acid, *β*-amyrin, and N-butylbenzene-sulfonamide [9, 20].

Moreover, the stem bark of *P. africana* has also been used in traditional medicine to treat several other diseases, such as benign prostatic hyperplasia, epilepsy, hemorrhage, arthritis, hypertension, and diarrhea [20, 21]. Unfortunately, the continued use of this plant constitutes a serious threat to wild populations. Indeed, the species was recently classified as an endangered species and, as such, the supply of stem bark is not sufficient to meet global demand [22]. To meet the global demand, we previously developed a micropropagation protocol for *P. africana* [23]. In this study, however, we examined the antiprostate cancer potential of the micropropagated juvenile six-month-old *P. africana* plants based on our protocol as an alternative to the use of the stem bark of wild *P. africana* plants using PC-3 cell line. In addition, we compared the chemical profiles of the different parts of the six-month-old plants and that of the bark of a mature wild *P. africana* plants using Fourier transform near-infrared (FT-NIR) spectrometry, gas chromatography-mass spectrometry (GC-MS), and high-performance liquid chromatography (HPLC). Thus, our study provides basis for the potential use of micropropagated *P. africana* in future drug development for prostate cancer treatment.

## 2. Material and Methods

### 2.1. Chemicals

Analytical-grade chemicals were used for HPLC analysis, including trifluoroacetic acid (Sigma-Aldrich, St. Louis, MO, USA), acetonitrile (Thermo Fisher Scientific, Oxford, UK), and ultrapure water produced using a Milli-Q system (Millipore, Burlington, MA, USA).

### 2.2. Plant Material Collection and Extract Preparation

Plant material used in this study consisted of stem bark samples (500 g) of wild *P. africana* provided by the Natural Chemotherapeutics Research Institute, Ministry of Health, Uganda. A voucher (specimen number KIOM201901022377) was deposited at the Korean Herbarium of Standard Herbal Resources (Index Herbarium code: KIOM) at the Korean Institute of Oriental Medicine (KIOM), Herbal Medicine Resources Research Center, South Korea.

**Figure 1:**
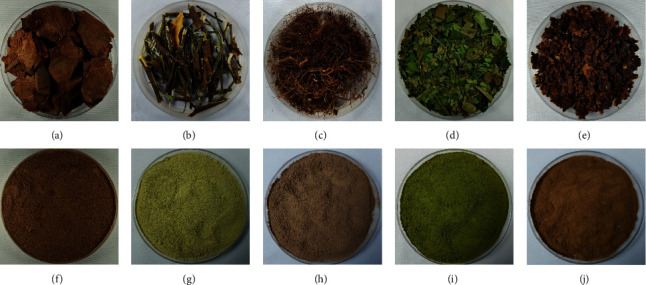
*Prunus africana* samples used in the experiment: (a) wild *P. africana* stem bark (PWS); (b) juvenile stem bark from cloned *P. africana* plant (PIS); (c) juvenile roots from cloned *P. africana* plant (PIR); (d) juvenile leaves from cloned *P. africana* plant (PIL); (e) in vitro callus from leaf explant (PC); (f) wild stem bark powder; (g) juvenile stem bark powder from cloned *P. africana* plant; (h) juvenile root powder from cloned *P. africana* plant; (i) juvenile leaves powder from cloned *P. africana* plant; (j) callus powder.

The following sample types were used: wild *P. africana* stem bark (PWS) (Figure 1(a)), stem of a six-month-old micropropagated *P. africana* plant (PIS) (Figure 1(b)), roots of a six-month-old micropropagated *P. africana* plant (PIR) (Figure 1(c)), leaves of a six-month-old micropropagated *P. africana* plant (PIL) (Figure 1(d)), and *P. africana* calluses generated from leaf explants (PIC) (Figure 1(e)). Each sample was ground to a fine powder (Figure 1(g)–1(j)) and then extracted and concentrated as previously described [24]. Vacuum dried extracts were used for antiprostate cancer assays.

### 2.3. High-Performance Liquid Chromatography (HPLC) Analysis of *P. africana*

Powdered PWS, PIS, PIR, PIL, and PIC samples (500 mg, each) were sequentially extracted twice for 30 min using 50 mL methanol and an ultrasonicator. Each extract was concentrated *in vacuo* using an evaporator, followed by dissolving the extract in methanol at 50 *µ*g/mL and filtration through a 0.22 *μ*m membrane filter (Whatman International Ltd., Maidstone, UK). Samples were then stored at 4°C until use. HPLC was performed using a Dionex UltiMate 3000 system (DAD; Thermo Fisher Scientific, CA, USA). Output signals from the detector were processed using Chromeleon software (v. 7). A total of 10 *μ*L of each sample was injected using an autosampler. Chromatographic separation was achieved using a Gemini C_18_ column (4.6 × 250 mm, 5 *μ*m; Phenomenex, Torrance, CA, USA) with the following mobile phases: 0.1% trifluoroacetic acid in water (v/v) as solvent A and acetonitrile as solvent B at a flow rate of 1 mL/min with a total run time of 50 min. HPLC elution conditions were optimized as follows: 0–2 min, 0%–3% B; 2–30 min, 3%–35% B; 30–31 min, 35%–50% B; 31–35 min, 50% B; 35–40 min, 50%–100% B; 40–45 min, 100% B; 45–50 min, 3% B; column oven temperature was 40°C, and detection wavelengths were 203, 254, 280, and 320 nm.

### 2.4. Gas Chromatography-Mass Spectrometry (GC-MS) Analysis of *P. africana*

Powdered PWS, PIS, PIR, PIL, and PIC samples (50 mg, each) were extracted using 1 mL 100% methanol and sonication for 30 min, followed by filtration through a 0.2 *μ*m syringe membrane filter (Whatman International Ltd). Analysis was performed using a 7890B GC-MS system (Agilent Technologies, Atlanta, GA, USA) coupled with a 7977B model mass detector (Agilent Technologies) and using a DB-5 MS capillary column (30 m × 0.25 mm × 0.25 *μ*m). Briefly, 1 *μ*L extract was injected in split mode at a ratio of 1/20 under the following chromatographic conditions: 250°C injection temperature and 50°C initial oven temperature, which was increased to 110°C over the next 5 min and then to 300°C at 7°C/min. A mass analyzer was used for scanning from 30 to 600 amu. Peaks were distinguished by comparison with experimental mass spectra at the National Institute of Standards and Technology (NIST) and Wiley GC-MS libraries.

### 2.5. Fourier Transform Near-Infrared (FT-NIR) Analysis of *P. africana*

Powdered PWS, PIC, PIR, PIL, and PIS samples (3 g, each) were placed in 22 mm vials and analyzed as previously described by Komakech et al. [23] using a TANGO FT-NIR spectrometer (Bruker Optics, Billerica, MA, USA).

### 2.6. Cancer Cell Line and Cell Culture Conditions

The PC-3 cell line was subcultured in tissue culture flasks containing Dulbecco's Modified Eagle Medium supplemented with 1% penicillin-streptomycin, 10% fetal bovine serum, and 1% nonessential amino acids. Cells were incubated in a CO_2_ incubator at 5% CO_2_ and 95% relative humidity. After trypsinization, cell counts were performed and cell viability was assessed using trypan blue staining and a hemocytometer. A known number of cells (2 × 10^3^ cells/well in 100 *μ*L medium) were seeded in 96-well microtiter plates for the methyl tetrazolium bromide (MTT) assay.

### 2.7. Treatment and MTT Cell Viability Assay of PC-3 Cells

Antiprostate cancer effects of PWS, PIC, PIR, PIL, and PIS extracts were determined *in vitro* using an MTT assay with PC-3 cells. To enhance cell attachment, the PC-3 cells were seeded at a known density in 96-well microtiter plates and incubated at 37°C at 5% CO_2_ and 95% relative humidity for 24 h. After incubation, extracts were added to the cells at concentrations of 0, 10, 30, 60, 90, or 270 *μ*g/mL. Doxorubicin was used as a positive control, and a blank control was included to which the same concentrations of fresh medium were added. The plates were incubated in a CO_2_ incubator for 48 h before aspirating the medium from each well. The cells were then washed using phosphate-buffered (PBS) solution before adding a fresh medium. A 30 *µ*L aliquot of MTT (5 mg/mL in PBS) was added to each well, followed by incubation at 37°C for 4 h. The medium was then aspirated, and dimethyl sulfoxide (DMSO) was added to solubilize any formazan crystals formed. After incubation with cell-counting kit solutions for 1 h, absorbance was measured at 450 nm using a microplate reader (Versa Max), and cell growth inhibition caused by each extract was expressed as the corresponding IC_50_ value.

### 2.8. Apoptosis and Viability Assays

Apoptosis induced by PWS and PIR extracts was determined by staining PC-3 cells with fluorescein isothiocyanate annexin-V and propidium iodide (PI) stains using the FITC Annexin V Apoptosis Detection Kit II (BD Biosciences, San Jose, CA, USA), according to manufacturer instructions. Cells were stained for 20 min on ice using the appropriate antibodies in a binding buffer (PBS with 2% FBS and 1 mM EDTA); analysis was performed using a fluorescence-activated cell sorter (FACS Canto II; BD Biosciences). Cell viability was determined using Cell Counting Kit-8 (CCK-8; Dojindo Molecular Technologies, Rockville, MD, USA). Briefly, 2 × 10^4^ cells/100 *μ*L were seeded in a 96-well plate, treated with PWS and PIR extracts at concentrations of 10, 30, 60, 90, and 270 *µ*g/ml and incubated at 37°C for 48 h. Then, 10 *μ*L CCK-8 was added to each well, and absorbance was measured at 450 nm using the SpectraMax i3x Multi-Mode Microplate Reader (Molecular Devices, San Jose, CA, USA).

### 2.9. Western Blot Analysis of PC-3 Cells after Treatment with *P. africana* Extracts

Proteins were extracted from PC-3 cells treated with different concentrations (0, 10, 30, 60, 90, and 270 *μ*g/mL) of PWS and PIR extracts using RIPA buffer (50 mM Tris-HCl, pH 7.4, 150 mM NaCl, 1% Triton X-100, 0.5% sodium deoxycholate, 0.1% SDS) (GenDEPOT, Baker, TX, USA). The cell lysate was separated on 8%–15% SDS-PAGE gels and transferred to PVDF membranes (Millipore, Bedford, MA, USA) treated with primary antibodies against cleaved caspase-3 (9661; Cell Signaling Technology, Danvers, MA, USA) and actin (sc-47778; Santa Cruz Biotechnology, Dallas TX, USA). After incubation with peroxidase-conjugated anti-rabbit or anti-mouse IgG (Jackson ImmunoResearch, West Gove, PA, USA), signals were detected using SuperSignal West Pico Chemiluminescent Substrate (Pierce). Western blots were visualized using WSE-6100 LuminoGraph (ATTO, Tokyo, Japan).

### 2.10. 4′,6-Diamidino-2-Phenylindole (DAPI) Staining

PC-3 cells were maintained in Roswell Park Memorial Institute (RPMI) 1640 medium supplemented with 10% (v/v) fetal bovine serum and antibiotics. Cells were seeded on an eight-well slide and incubated overnight. After 24 h, cells were treated with respective IC_50_ concentrations of PWS (52.30 *μ*g/ml) and PIR (82.40 *μ*g/ml), negative control cells were treated with DMSO (0.1%), and positive control cells treated with doxorubicin (1.13 uM) and incubated for 48 h. Incubated cells were washed with PBS, fixed with 2% paraformaldehyde and stained with DAPI (1 *μ*g/mL), and incubated for 5 min. Cells placed on slides were observed under a fluorescence microscope (Olympus CKX53; Olympus, Tokyo, Japan). The criteria defining apoptosis of DAPI staining, such as nuclear pyknosis and fragmentation, were applied as described in a previous study [25]. The number of nuclei showing these morphological characteristics was counted as DAPI positive cells under the immunofluorescence microscope at 40× magnification. For each sample, three independent areas of interest with at least 1000 total nuclei per area were counted using Image J software and DAPI positive cells were showed as % of total cells. Statistical analyses were performed using a one-way analysis of variance (ANOVA) with GraphPad Prism 8.4.3.

### 2.11. Statistical Analysis

Means, standard error of the means, and proportions were determined. LC_50_ values were determined using linear regression. The data were analyzed by ANOVA, and differences were tested using Bonferroni's post hoc test implemented in GraphPad Prism software (ver. 5. 03). Statistical significance is reported at *p* < 0.05. FT-NIR spectroscopy multivariate statistical analyses were performed, and Ward's algorithm was calculated using OPUS TANGO-R software for homogeneity cluster analysis.

## 3. Results

### 3.1. High-Performance Liquid Chromatography Analysis of *P. africana*

**Figure 2:**
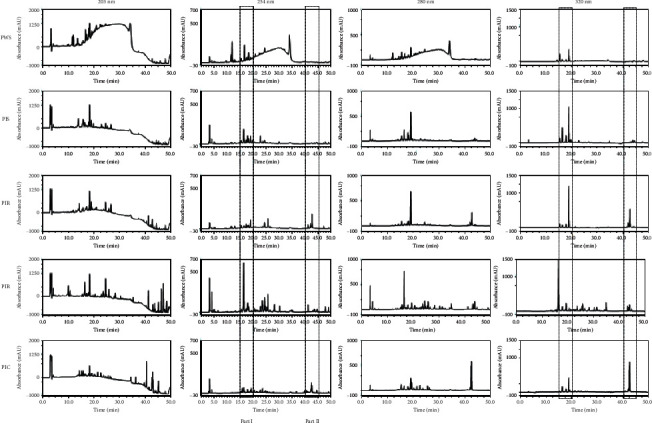
Finger-printing analysis of the extracts derived from *P. africana* by the HPLC-DAD method. PWS, mature *P. africana* stem bark; PIS, stem bark of juvenile cloned *P. africana*; PIR, root of juvenile cloned *P. africana*; PIL, leaves of juvenile cloned *P. africana*; PIC, callus generated from leaf explant of *P. africana.* Part I: retention part, 15–20 min; Part II: retention part, 40–45 min.

Finger-printing analysis results of *P. africana* extract are shown in Figure 2. Sufficient selectivity and separation were recorded at 254 and 320 nm, respectively. Relative to PWS, sample PIS showed comparable component patterns at 15–20 min (Part I) and 40–45 min (Part II), although peak intensities differed. The components of sample PIC were comparable to those of sample PIR; in particular, these profiles appeared to be quite similar in Part I and Part II of the chromatogram at 320 nm. Among the five different *P. africana* sample types, sample PIL showed the most complex pattern, with various component profiles at all applied UV wavelengths.

### 3.2. Gas Chromatography-Mass Spectrometry Analysis of *P. africana*

**Figure 3:**
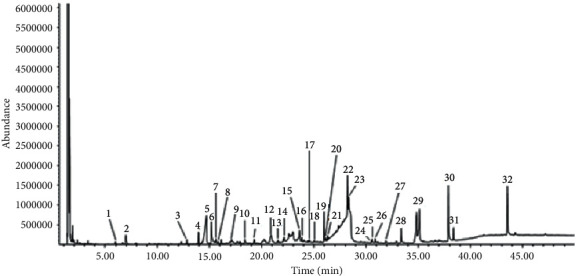
GC/MS chromatogram of the methanol extract of sample PWS.

Based on mass spectra, retention times, and quality ratio analysis, GC-MS analysis results for PWS extract indicated 32 components (Figure 3 and Table 1). Identified compounds included benzoic acid (14.02%), *β*-sitosterol (8.37%), 13-docoseamide (6.49%), and n-hexadecanoic acid (4.95%). Comparative analyses of the other extracts were performed based on the chemical composition of PWS extract. According to mass spectra and retention times (Table 1), 4H-pyran-4-one,2,3-dihydro-3,5-dihydroxy-6-methyl- (**4**), benzoic acid (**5**), 5-hydroxymethyl-2-furaldehyde (**8**), n-hexadecanoic acid (**23**), 9,12-octadecadienoic acid (Z, Z)- (**24**), octadecanoic acid (**26**), 9-octadecenamide, (Z)- (**28**), 13-docosenamide, (Z)- (**30**), and *β*-sitosterol (**32**) were present in all extracts. Among these nine components, **(5)** represented the largest proportion of all compounds detected in a callus extract, compared with the PWS extract. In contrast, five components, including 3,4-altrosan (**12**), benzenepropanol, 4-hydroxy-3-methoxy- (**15**), benzaldehyde, 4-hydroxy-3, 5-dimethoxy- (**16**), 4-(hydroxymethyl)-2,6-dimethoxyphenol (**17**), and 6-hydroxy-5-trifluoromethylcyclohexa-1,3-diene (**19**), occurred only in PWS extracts. Vanillic acid (**14**) and (R)-alpha-(*β*-D-glucopyranosyloxy) benzene-acetonitrile (**29**) were detected in PWS and PIS extracts.

### 3.3. Fourier Transform Near-Infrared Analysis of *P. africana*

**Figure 4:**
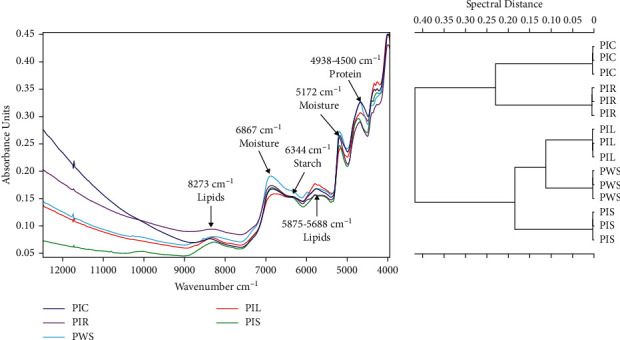
Multivariate statistical analysis of different samples obtained from *in vitro* regenerated and wild *Prunus africana* plants. (a) TANGO FT-NIR spectroscopy analysis results (wave number frequency range = 12,000–4,000 cm^−1^). (b). FT-NIR chemical characterization based on Ward's algorithm clustering dendrogram (data preprocessing-first derivative + vector normalization; standard (Euclidean distance); frequency range = 12,000–4,000 cm^−1^). PIC, *P. africana* callus sample; PIR, root sample obtained from *in vitro* regenerated *P. africana* plant; PIL, leaf sample obtained from *in vitro* regenerated *P. africana* plant; PWS, stem sample obtained from mature wild *P. africana* plant; PIS, stem sample obtained from *in vitro* regenerated *P. africana* plant.

FT-NIR analyses of PWS, PIC, PIR, PIL, and PIS samples showed six prominent FT-NIR peaks within the region between 8,500 and 4,000 cm^−1^, which included peaks at 8,273, 6,867, 6,344, 5,875–5,688, 5,172, and 4,938–4,500 cm^−1^ (Figure 4(a)). The vertical scale of the dendrogram (Figure 4(b)) indicates the numerical distance between PWS, PIC, PIR, PIL, and PIS; as can be seen, all samples showed high heterogeneity at a distance of approximately 0.45. However, PIS, PIL, and PWS showed higher similarity and clustered with a spectral distance (heterogeneity) of 0.18, and further separation was observed in which PIL and PWS showed a closest spectral distance (heterogeneity) of approximately 0.12. Similarly, PIC and PIR clustered with a spectral distance (heterogeneity) of approximately 0.24.

### 3.4. *In Vitro* Anticancer Effects of *P. africana* Extracts on PC-3 Cancer Cells

**Figure 5:**
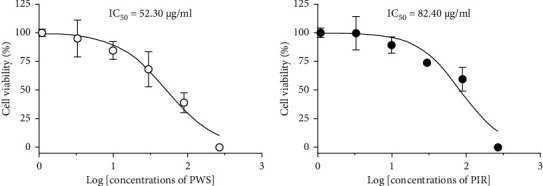
CCK-8 assay of PC-3 cells viability when exposed for 48 h at different concentrations of PWS and PIR samples. Results were expressed as percentage of cell viability and each point expressed as mean ± SD (*N* = 3). IC_50_ values are calculated by using GraphPad Prism software.

A preliminary test failed to show any remarkable concentration-dependent antiprostate cancer effects from PIS, PIL, or PIC on PC-3 cell lines, whereby they were excluded from the main experiment. In contrast, PWS and PIR did show dose-dependent antiprostate cancer effects on PC-3 cells after exposure for 48 h. Therefore, we determined cancer cell viability using the CCK-8 assay and obtained IC_50_ values of 52.30 and 82.40 *μ*g/mL for PWS and PIR, respectively (Figure 5).

### 3.5. Annexin-V and PI Staining

**Figure 6:**
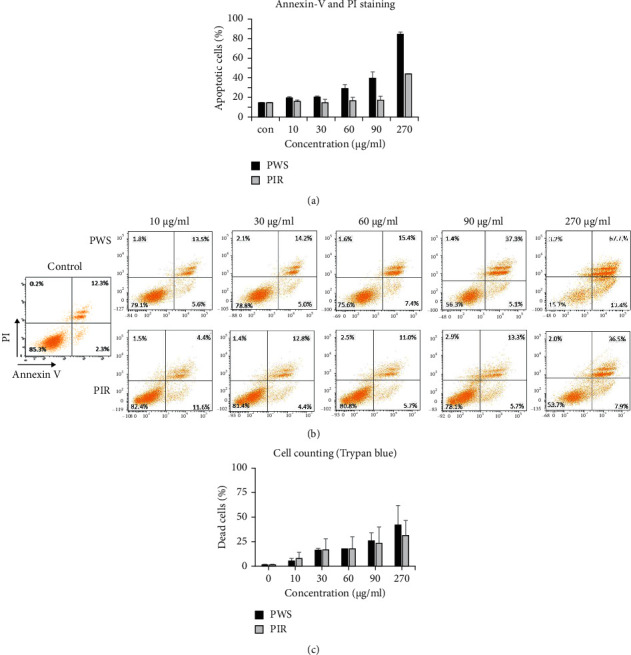
Apoptosis assay of PC-3 cells after 48 h treatment with PWS and PIR samples. (a) Apoptosis levels after annexin-V and PI staining of treated PC3-cells. (b) Apoptosis levels of treated PC-3 cells by flow cytometry (FACS) after annexin-V and PI staining (the upper left quadrant indicates necrotic cells (annexin V (−)/PI (+)), the upper right quadrant indicates late apoptotic cells (annexin V (+)/PI (+)), lower right quadrant indicates early apoptotic cells (annexin V (+)/PI (−)), and the lower left quadrant indicates healthy cells (annexin V (−)/PI (−)). (c) Dead cells of treated PC-3 cells when stained with trypan blue.

To confirm that PWS and PIR induced apoptosis in PC-3 cells, we examined apoptosis levels after incubation with PWS and PIR extracts for 48 h using FACS, followed by annexin-V and PI staining. PWS and PIR induced apoptosis in PC-3 cells in a dose-dependent manner. Specifically, treatment with PWS produced a higher proportion of apoptotic cells than the PIR treatment (Figure 6(a)). The proportion of apoptotic cells correlated positively with extract concentration; furthermore, PWS at 90 *μ*g/mL resulted in 37.3% apoptotic cells, compared with 13.3% caused by PIR treatment (Figure 6(b)). A similar trend was observed for trypan blue staining, which showed more dead cells under the PWS treatment than under the PIR treatment (Figure 6(c)).

### 3.6. 4′,6-Diamidino-2-Phenylindole (DAPI) Staining

**Figure 7:**
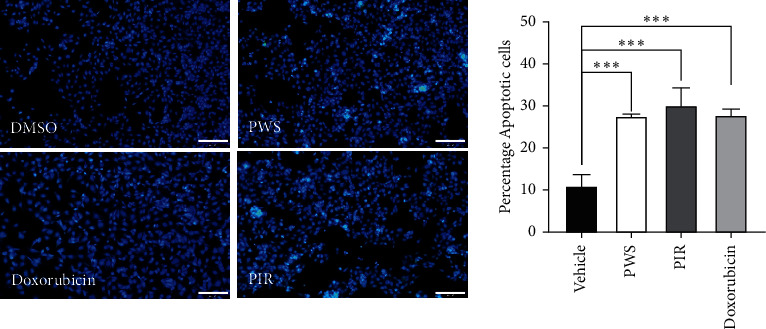
Changes in PC-3 cells nuclear condensation observed after DAPI staining when treated with PWS (52.30 *μ*g/ml) or PIR (82.40 *μ*g/ml), vehicle (0.1% DMSO), and doxorubicin (1.13 uM) as a positive control and incubated for 48 h. Representative image and percentage of apoptotic cells were shown. ^*∗∗∗*^*p* < 0.001 compared with vehicle-treated controls.

The proportion of DAPI-stained apoptotic PC-3 cells following treatment with DMSO, PWS, PIR, and doxorubicin was 10.5%, 27%, 28%, and 27.5%, respectively (Figure 7). Furthermore, DAPI (4′,6-diamidino-2-phenylindole) staining showed significant (*p* < 0.001) induction of cell death after treatment with PWS, PIR, or doxorubicin (positive control), compared with the negative control, with no significant difference observed among PWS, PIR, or doxorubicin-treated cells.

### 3.7. Western Blot Analysis

**Figure 8:**
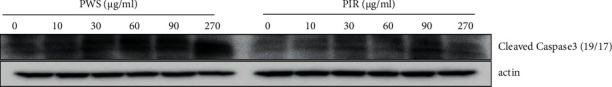
Western blot analysis of PC-3 after 48 h treatment with PWS and PIR samples.

Caspase-3 activation was determined by western blotting of cleaved caspase-3. PWS treatment induced higher cleaved caspase-3 levels in PC-3 cells than PIR treatment, an indication that PWS induced substantial apoptosis in PC-3 cells compared with PIR (Figure 8).

## 4. Discussion

FT-NIR spectrometry has been used in numerous studies to characterize chemicals present in samples [23, 26]. The absorption band at 8,273 cm^−1^ in the current study was due to the second C–H overtone produced by lipids; in turn, the peak at 6,867 cm^−1^ was due to the first O–H overtone caused by moisture, while the peak at 6,344 cm^−1^ was due to the first O–H overtone linked to starches; additionally, the peak at 5,875–5,688 cm^−1^ was due to the first C–H overtone linked to lipids; in turn absorbance peaks between 4,938 and 4,500 cm^−1^ resulted from the combination of N–H, O–H, and C–H stretching associated with proteins [26–29]. Therefore, the similarity in the peaks of these samples associated with specific functional groups offers an indication of the high degree of chemical homogeneity among PWS, PIS, PIR, PIL, and PIC samples. Ward's algorithm clustering has been commonly used to characterize samples [30]. The dendrogram constructed herein showed high heterogeneity among PWS, PIS, PIR, PIL, and PIC samples, presumably due to higher similarity of the various near-infrared spectra generated by the respective sample. As shorter distances between samples indicate close chemical phylogenetic relationships, the similarity of these samples regarding antiprostate cancer effects may be due to the similarity in chemical composition among different *P. africana* samples.

In recent years, plant-derived phytochemicals have been widely used as chemopreventive and chemotherapeutic agents for treating various cancers, including prostate cancer [31]. In this study, numerous phytochemicals present in *P. africana* extracts were identified (Table 1), and antiprostate cancer effects of the *P. africana* samples examined may be associated with some of these compounds. *β*-Sitosterol is one of the phytochemicals contributing to the antiprostate cancer effects of *P. africana* [18] and has been observed to induce apoptosis in human prostate cancer cells in cases of prostate lymph-node carcinoma [32, 33].

Nuclear factor kappa-B (NF-*κ*B) inhibition is crucial in the arrest of cancer cell growth [34], and it exerts antimutagenic effects [35]. Therefore, the presence of 2,3-dihydro-3,5-dihydroxy-6-methyl-4H-pyran-one in *P. africana* extracts may be responsible for the antiproliferative and proapoptotic effects of the different extracts on PC-3 cancer cells due to its ability to inactivate NF-*κ*B. Additionally, the presence of benzoic acid in *P. africana* extracts may also be responsible for its anticancer effects. Indeed, previous studies revealed that benzoic acid derivatives delayed prostate cancer-cell growth, thereby preventing the expression of oncogenes by inhibiting histone deacetylases [36].

Annexin-V and PI staining are important techniques for accurate assessment of cell death [37]. Hence, to determine whether the growth inhibitory effect of PWS and PIR on PC3 cells was associated with the induction of apoptosis, the cells were stained with Annexin V-FITC and propidium iodide. Annexin V is a calcium-dependent phospholipid-binding protein with a high binding affinity for phosphatidylserine-phospholipid, which is exposed at the outer layer of the cell membrane during the initial stages of apoptosis [38, 39]. Conjugation of a fluorophore to Annexin V (FITC) therefore allows for detection of early apoptotic cells. Also, as apoptosis progresses, the cell membrane loses integrity and cells become necrotic. At this stage, propidium iodide—a fluorescent viability dye, which is impermeable to the cell membrane—diffuses freely into dying cells. Therefore, staining of cells with annexin V in conjunction with PI is usually done to establish the integrity of the cell membrane and distinguish living cells from both early and late apoptosis [40]. In the present study, annexin-V and PI staining showed significant induction of PC-3 cell death after treatment with PWS and PIR extracts. As depicted in Figure 6, exposure of cells to PWS and PIR increased the proportion of apoptotic cells (both early and late apoptosis) in comparison with negative control cells, although PWS displayed higher apoptosis-inducing capacity compared with PIR. The extracts displayed different chemical profiles. Some of the major compounds identified in PWS were either absent or only found as minor constituents in PIR. This may partly account for the increased apoptosis-inducing effects of PWS since some of these bioactive compounds (e.g., squalene and vanillic acid) are known to induce apoptosis in cancer cells.

DAPI is a DNA-specific fluorochrome that binds strongly to adenine-thymine-rich regions of DNA and is widely used to analyze nuclear morphologic changes such as DNA fragmentation during apoptosis [41]. In the present study, the nuclear features of the cells were examined by DAPI staining. DAPI is known to only inefficiently pass through an intact cell membrane and hence preferentially stain dead cells [42] including dead PC-3 cells [43]. As depicted in Figure 7, data from this study showed that nuclei of the negative control cells were homogeneously stained with DAPI and displayed less blue fluorescence intensity due to the presence of more intact cell membrane compared with the higher staining intensity of the cells treated with PWS, PIR, and doxorubicin, which suggest a compromised apoptotic cell membrane. PC-3 cells treated with PWS, PIR, and doxorubicin (positive control) showed altered nuclear DNA staining, nuclear fragmentation, and condensation. In fact, the DAPI staining is an indication of PC-3 cell death via apoptosis [44].

In a previous *in vitro* anticancer activity study, *P. africana* stem bark methanolic and aqueous extracts had respective IC_50_ values of 24.4 ± 3.6 and 19.9 ± 0.9 *µ*g/ml against DU-145 prostate cancer cell lines [45]. In the same study, when these same extracts were tested against 22RV1 prostate cancer cell lines, the IC_50_ values were 19.6 ± 5.8 and 20.7 ± 0.8 *µ*g/ml for methanolic and aqueous extracts, respectively [45]. Compared with our findings, the IC_50_ values greatly differed. The differences in the IC_50_ values could be attributed to differences in the prostate cancer cell lines and extractants used. A study by Ghagane et al. [46] demonstrated that indeed the type of extracting solvent significantly influenced growth inhibition levels of PC-3 and DU-145 prostate cancer cell lines and thus the IC_50_ values. Another study by Yesil-Celiktas et al. [47] showed that different cancer cell lines were inhibited to varying degree by plant extracts.

Caspases are responsible for cell apoptosis, and specifically, caspase-3 activation plays a critical role in the execution of all apoptosis signaling pathways [48, 49]. Therefore, the detection of elevated levels of cleaved caspase-3 by western blotting following PWS and PIR treatment is a strong indication of apoptotic cell death in PC-3 cells via the intrinsic apoptosis pathway. In a previous *in vitro* study, the stem bark extract of *P. africana* was also observed to exhibit significant apoptosis in the PC-3 cell line and lymph node carcinoma of the prostate (LNCaP) [18].

## 5. Conclusion

PIR extracts exhibited a dose-dependent, *in vitro* antiprostate cancer effect on PC-3 prostate cancer cells similar to that of the traditional PWS extract. Therefore, these extracts can be used as an alternative to stem bark collected from wild populations of *P. africana*, which may contribute to mitigating the threat of overexploitation of this endangered species. Furthermore, this study provides a sound theoretical basis for further research on the development of new prostate-cancer drugs from micropropagated *P. africana* plants.

## Figures and Tables

**Table 1:** Phytochemical components detected in the methanol extract of *P. africana* experimental materials by GC/MS analysis. *t*_*R*_: retention time (min); %: percent of total.

No.	Identified compound	PWS		PIS		PIR		PIL		PC	
		*t*_*R*_^1^	%2	*tR*	%	*tR*	%	*tR*	%	*tR*	%
1	3-Furanmethanol	5.96	0.39	—	—	—	—	5.97	0.16	5.97	0.79
2	Dihydroxyacetone	7.01	2.22	6.96	0.72	—	—	7.01	0.62	7.10	1.31
3	Benzoic acid, methyl ester	12.83	0.46	—	—	—	—	8.11	0.35	—	—
4	4H-Pyran-4-one,2,3-dihydro-3,5-dihydroxy-6-methyl-	13.95	1.13	13.95	0.63	13.94	0.66	9.19	0.06	14.00	2.87
5	Benzoic acid	14.68	14.02	14.60	3.10	14.55	1.83	12.83	0.15	14.48	0.71
6	Catechol	15.17	4.74	—	—	15.17	0.32	13.97	0.80	—	—
7	4-Vinylphenol	15.61	0.77	15.62	0.65	15.63	0.80	14.99	6.79	—	—
8	5-Hydroxymethyl-2-furaldhyde	15.81	0.31	15.81	0.81	15.81	0.11	15.20	0.80	16.03	24.79
9	Isosorbide	17.10	0.98	—	—	—	—	15.62	1.66	—	—
10	Phenol, 2,6-dimethoxy-	18.39	0.41	—	—	18.39	0.04	15.83	0.32	—	—
11	4-Hydroxy-3-methoxybenzaldehyde	19.30	0.41	25.03	0.14	—	—	—	—	—	—
12	3,4-Altrosan	20.84	3.30	—	—	—	—	—	—	—	—
13	Mandelamide	21.54	0.40	—	—	—	—	21.64	1.41	—	—
14	Vanillic acid	22.13	0.93	—	—	22.12	0.78	—	—	—	—
15	Benzenepropanol, 4-hydroxy-3-methoxy-	23.61	2.14	—	—	—	—	—	—	—	—
16	Benzaldehyde, 4-hydroxy-3,5-dimethoxy-	23.83	0.75	—	—	—	—	—	—	—	—
17	4-(Hydroxymethyl)-2,6-dimethoxyphenol	24.57	0.17	—	—	—	—	—	—	—	—
18	(E)-4-(3-Hydroxyprop-1-en-1-yl)-2-methoxyphenol	25.03	0.18	25.03	0.14	25.03	0.10	25.05	0.38	—	—
19	6-Hydroxy-5-trifluoromethylcyclohexa-1,3-diene	25.98	3.65	—	—	—	—	—	—	—	—
20	Benzoic acid, 4-hydroxy-3,5-dimethoxy-	26.13	0.29	26.12	0.08	26.20	0.13	—	—	—	—
21	Isopropyl myristate	26.31	0.40	26.31	0.15	—	—	—	—	—	—
22	Sorbitol	27.95	0.05	27.00	0.09	29.62	5.75	29.82	2.88	—	—
23	n-Hexadecanoic acid	28.21	4.95	28.22	2.34	28.22	3.72	28.24	1.52	28.23	3.18
24	9,12-Octadecadienoic acid (Z, Z)-	30.51	0.20	30.52	0.79	30.52	0.64	30.57	0.88	30.52	0.21
25	Oleic acid	30.58	0.76	30.60	0.99	—	—	—	—	30.60	1.72
26	Octadecanoic acid	30.87	0.47	30.87	0.66	—	—	30.89	0.58	30.88	0.69
27	Benzyl, beta-d-glucoside	31.90	0.32	31.90	0.17	—	—	32.05	0.28	31.97	0.16
28	9-Octadecenamide, (Z)-	33.35	1.95	33.35	0.80	33.35	1.05	33.36	0.33	33.35	0.59
29	(R)-alpha-(beta-D-glucopyranosyloxy)benzene-acetonitrile	35.10	6.60	—	—	35.10	1.91	—	—	—	—
30	13-Docosenamide, (Z)-	37.88	6.49	37.88	6.76	37.88	4.53	37.88	0.93	37.87	1.07
31	Squalene	38.38	1.09	—	—	—	—	38.40	5.34	—	—
32	Beta-sitosterol	43.55	8.37	43.57	16.77	43.56	7.75	43.56	4.76	43.55	4.02

## Data Availability

The data for this current study are available from the corresponding author upon reasonable request.
